# Predicting Factors for Allogeneic Blood Transfusion and Excessive Postoperative Blood Loss after Single Low-Dosage Intra-Articular Tranexamic Acid Application in Total Knee Replacement

**DOI:** 10.1155/2017/2729487

**Published:** 2017-02-26

**Authors:** Paphon Sa-ngasoongsong, Siwadol Wongsak, Noratep Kulachote, Pongsthorn Chanplakorn, Patarawan Woratanarat, Viroj Kawinwonggowit

**Affiliations:** Department of Orthopaedics, Faculty of Medicine, Ramathibodi Hospital, Mahidol University, Salaya, Thailand

## Abstract

*Background.* Recently, intra-articular tranexamic acid (IA-TXA) application has become a popular method for perioperative blood loss (PBL) reduction in total knee replacement (TKR). Nevertheless, through our knowledge, no previous studies had shown the correlation perioperative factors and the risk of excessive PBL or need of blood transfusion (BT) after IA-TXA.* Materials and Methods.* A retrospective study was conducted in patients underwent 299 primary TKRs, using IA-TXA, during 2-year period (2013-2014). Patient's characteristic and perioperative data were reviewed and collected. PBL was measured as total hemoglobin loss (THL), estimated total blood loss (ETBL), and drainage volume per kg (DV/kg). Excessive PBL was defined as PBL that exceeded 90th percentile.* Results*. From multivariate analysis, low preoperative hemoglobin (Hb) level and body mass index (BMI) were the significant predictors of postoperative BT (*p* < 0.0001 and 0.003, resp.). Excessive THL significant associated with preoperative Hb (*p* < 0.0001). Excessive ETBL significantly associated with preoperative Hb, height, preoperative range-of-motion, and creatinine clearance (*p* < 0.05 all). Low BMI and large prosthesis size were the significant predictors of excessive DV/kg (*p* = 0.0001 and 0.002, resp.).* Conclusions*. Low preoperative Hb and BMI were the significant risks of postoperative transfusion after TKR with IA-TXA. Moreover, multiple perioperative factors could result in higher PBL.

## 1. Background

Intra-articular tranexamic acid (IA-TXA) application has recently become one of the most popular blood conservative methods in total knee replacement (TKR) due to its successfully proven effectiveness on reduction of postoperative blood loss (PBL) and blood transfusion (BT) without significant risk of postoperative complication [1–7]. Nonetheless, even with our best knowledge, of using this new technique, the amount of PBL is sometimes high and BT may be required, while these incidences seem to vary in different report studies, as 300–1,300 mL of PBL and 0%–20% of BT [8–13]. Although there have been numerous studies that showed predictors of blood loss and transfusion requirement after TKR (such as age, gender, body mass index, preoperative hemoglobin (Hb) level, use of postoperative anticoagulation, surgical time, and intraoperative blood loss [14, 15]), the local application of tranexamic acid (TXA), which directly controls surgical bleeding, from raw surfaces and small noncoagulated blood vessels, might have different effects on these predicting factors of blood transfusion requirement presented in the previous literatures. Moreover, the risk factors associated with BT or PBL after using IA-TXA application have not yet been studied, and we thought that identifying patients with increased risk of considerable PBL and the need for perioperative BT is an important step toward establishing a better effective blood management strategy [16]. Therefore, this study aimed to identify the preoperative risk factors among the patients undergoing TKR with IA-TXA application which correlated with the substantial amount of postoperative blood loss and the need for postoperative blood transfusion. We hypothesized that some perioperative factors would influence the needs of postoperative transfusion and blood loss after using IA-TXA.

## 2. Methods

### 2.1. Study Design, Participants, and Inclusion and Exclusion Criteria

This was a singled-centered retrospective study and was approved by our institutional review board (protocol number 02-58-78). The electronic hospital database was used to identify the patients who underwent total joint replacement surgery from January 2013 to December 2014. The inclusion criteria were the patients who underwent primary total knee replacement, receiving operation from only one surgeon (VK) with the same surgical technique, prosthesis, and using IA-TXA application protocol the same as our previous study [17]. The exclusion criteria were patients who had simultaneous bilateral TKR or revision knee surgery, patients undertaking anticoagulant medication, and cases with acquired or congenital bleeding disorders.

### 2.2. Surgical Procedure, IA-TXA Administration, and Perioperative Protocol

The surgical approach was medial parapatellar arthrotomy with midvastus approach. The anesthetic technique, either general or spinal anesthesia, was based on the decision of anesthesiologist, who was not involved in this study. The prosthesis was Nexgen® total knee system [Zimmer Inc., Warsaw, Indiana, USA] with patellar resurfacing in all patients, all prostheses were inserted with full cementation (Palacos, Heraeus Medical GmbH, Germany). A standard no. 8 drain tube was placed inside the knee joint and connected with high-pressure vacuum drain system (Drainobag® 600V Lock, B-Braun, Melsungen, Germany). The prepared IA-TXA solution, as 500 mg of TXA with a total of 25 ml volume, was then injected into the knee joint via drain tube after fascial and skin closure and application of compressive dressing. The drain was clamped for 2 hours before tourniquet deflation and then fully opened at ward. Postoperative care and transfusion guideline were the same as our previous study [17].

### 2.3. Data Collection and Outcome Measurement Method

The charts were reviewed, by using our electronic hospital database, to obtain required data including the patients' demographic data, perioperative data, and postoperative complications. Demographic data such as age, gender, weight, height, comorbid diseases and concurrent medications, preoperative knee osteoarthritis [OA] condition (as femorotibial angle measuring from standard long standing knee anteroposterior radiograph, knee osteoarthritis staging regarding Kellgren and Lawrence [KL] classification [18], and preoperative range-of-motion [ROM]), American Society of Anesthesiologist (ASA) physical status, and preoperative laboratory values (hemoglobin [Hb], platelet count, activated partial prothrombin time [aPTT], international normalized ratio [INR], albumin and creatinine clearance [CrCl]) were collected. Body mass index (BMI) and Charlson Comorbidity Index (CCI) were calculated. Perioperative and postoperative data, as side of operation, type of anesthesia, operative time, prosthesis size, intraoperative blood loss, drainage volume, hemoglobin on the 3rd postoperative day, amount of blood transfusion, and postoperative complications were recorded. Following the blood transfusion protocol from ASA guideline, transfusion was considered, when Hb was less than 8 gm% or the patient had positive anemic symptom (dyspnea, tachypnea, and hypoxemia) [19].

PBL was measured in 3 types: total hemoglobin loss (THL), estimated total blood loss (ETBL), and drainage volume per kg (DV/kg). THL and ETBL were calculated using the specific formulae [20–22]. DV/kg was defined as total drainage volume divided by patient's weight in kg. The definition of excessive PBL was by any type of PBL exceeding the 90th percentile cut-off point.

### 2.4. Statistical Analysis

Stata software version 11.0 (Stata Corp, College Station, Texas, USA) was used to analyze data. Continuous data and categorical data were presented as mean with standard deviation and number of cases with proportion. The association between each of the variables and blood transfusion or any types of excessive PBL was analyzed with univariate logistic regression analysis. Then the predicting factors with *p* < 0.15 were entered into multivariate logistic regression analysis. Significant value was defined as *p* < 0.05.

## 3. Results

### 3.1. General (Characteristic and Correlation with Blood Loss)

Between January 2013 and December 2014, a total of 251 patients with 299 primary unilateral total knee replacements (TKRs) (203 patients with unilateral TKRs and 48 patients with bilateral sequential TKRs), using intra-articular tranexamic acid (IA-TXA) application, were enrolled consecutively in this study. Among these patients, 208 of them were female (83%), and the average patients' age ± standard deviation was 69 ± 8 years. The incidence of blood transfusion was 5.4% (16 TKRs). Regarding the patients received transfusion, 15 of them (94%) were transfused only 1 unit, while one case (6%) needed 2 units of packed red cell transfusion. The mean THL, ETBL, and DV/kg was 1.6 ± 0.9 g/dL, 196 ± 122 mL, and 6.3 ± 3.5 mL/kg, respectively. The 90th percentile THL, ETBL, and DV/kg was 2.7 g/dL, 348.6 mL, and 10.6 mL/kg.

Table 1 demonstrated the characteristics of all 299 TKRs and the subgroup of patients based on the need of postoperative blood transfusion. There was a significant difference in preoperative factors, which were weight, BMI, preoperative Hb, INR, serum albumin, and CrCl level, between blood transfusion (BT) group and non-BT group (*p* < 0.05 all). Every postoperative blood loss (PBL) outcome in the BT group, including THL, ETBL, and DV/kg, was also significantly higher than the non-BT group (*p* < 0.05 all).

Table 2 showed the results of univariate logistic regression analysis for the effect of preoperative factors on BT and each type of excessive PBL. The significant preoperative risk factors for receiving BT and having excessive PBL from multivariate logistic regression analysis were illustrated in Table 3.

### 3.2. Risk Factors for Allogeneic Blood Transfusion

By univariate analysis, the preoperative factors that were significantly associated with BT were weight, BMI, preoperative ROM, Hb, INR, creatinine clearance and albumin (*p* < 0.15 all) (Table 2). However, multivariate regression analysis demonstrated that only preoperative Hb and BMI were the significant predictors for receiving postoperative BT (Hb; odds ratio [OR] = 0.23, 95% confidence interval [CI] = 0.12–0.43, *p* < 0.0001, and BMI; OR = 0.77, 95% CI = 0.65–0.91, *p* = 0.003) (Table 3). The area under the curve (AUC) of this prediction model was 0.896 (95% CI = 0.856–0.928).

### 3.3. Risk Factors for Excessive PBL

*THL > 2.7 g/dL*. The significant preoperative factors, from univariate analysis, were preoperative Hb and platelet count (*p* ≤ 0.0001 and 0.118, resp.) (Table 2). Multivariate analysis indicated that only preoperative Hb was the independent risk factor (OR = 2.23, 95% CI 1.56–3.18, *p* < 0.0001) (Table 3). The AUC was 0.756 (95% CI = 0.704–0.804).

*ETBL > 348.6 mL*. The significant preoperative factors, from univariate analysis, were age, male gender, weight, height, preoperative ROM, Hb, creatinine clearance, and large prosthesis (*p* < 0.15 all) (Table 2). Multivariate analysis demonstrated that the significant independent predictive factors were height (OR = 1.11, 95% CI 1.05–1.17, *p* = 0.0002), preoperative Hb (OR = 1.65, 95% CI 1.15–2.39, *p* = 0.007), preoperative ROM (OR = 1.03, 95% CI 1.00–1.06, *p* = 0.02), and CrCl (OR = 1.02, 95% CI 1.01–1.04, *p* = 0.006) (Table 3). The AUC was 0.804 (95% CI 0.754–847).

*DV/kg > 10.6 mL/kg*. The significant preoperative factors, from univariate analysis, were male gender, weight, BMI, preoperative ROM, platelet count, CrCl, albumin, and large prosthesis (*p* < 0.015 all) (Table 2). Multivariate analysis demonstrated that the significant independent predictive factors were BMI (OR = 0.78, 95% CI 0.69–0.88, *p* = 0.0001) and large prosthesis (OR = 3.91, 95% CI 1.67–9.14, *p* = 0.002) (Table 3). The AUC was 0.782 (95% CI 0.731–0.827).

## 4. Discussion

The result from this study showed that the significant independent risk factors for BT were lower preoperative Hb and lower BMI (*p* ≤ 0.0001 and 0.003, resp.), which was consistent with the previously published reports on TKR without IA-TXA application [13–15, 23–27].

Regarding the risk factors for excessive PBL, the excessive THL (>2.7 g/dL) was significantly associated with only higher preoperative Hb (*p* < 0.0001), and the excessive ETBL (>348.6 mL) was significantly associated with the patients with greater height, preoperative Hb, preoperative ROM, and CrCl (*p* < 0.05 all), whereas excessive DV/kg (>10.6 mL/kg) was significantly associated with lower BMI and larger prosthesis size (*p* = 0.001 and 0.002, resp.).

The variation in significant predicting factors on each type of PBL in our study might be explained by the different methods of outcome measurement (THL, ETBL, and DV/kg) and their correlation on the effect of blood loss reduction from IA-TXA application. The significant association of higher preoperative Hb on excessive THL or ETBL could be explained by the effect of IA-TXA and drain clamping method on initial phase of blood loss. As a result from our previous studies, IA-TXA application with 2-hour drain clamping significantly reduced the drainage blood loss rate in the first 4 hours after clamp release [17]. The drainage volume during this period was the initial bleeding from surgical bed into the knee joint after tourniquet release which had the Hb concentration nearly the same as the patients' Hb and considered as the majority of postoperative THL. Therefore, the THL from patients with higher preoperative Hb would be greater than those with lower preoperative Hb considering that the blood volume loss from initial clot formation was nearly the same between individual subjects. Consequently, higher preoperative Hb would directly associate with the greater PBL outcomes which use the difference of Hb or Hct concentration, such as THL and ETBL.

The effect of height, preoperative ROM, and CrCl on ETBL might be explained by the following reasons. The patients with greater height had higher estimated blood volume and greater ETBL would be a result from the specific formula [20–22]. Those with greater preoperative ROM would have lesser tightness of knee joint capsule and soft tissue, which resulted in larger knee volume after fascia closure. Therefore, the IA-TXA concentration in those with greater preoperative ROM would be lower than those with lesser preoperative ROM, which subsequently reduced the effect of blood loss reduction from this method. The effect of higher CrCl on ETBL might be explained by the better ability of TXA clearance in the patients' blood after systemic absorption [28] which resulted in lower serum TXA level and, therefore, decreased the effect of blood loss reduction, compared with the patients who had lower CrCl. However, the effect of these factors on ETBL was still minimal, as 2%–11% higher in ETBL, compared with the effect of higher preoperative Hb on ETBL as shown in Table 3.

The effect of lower BMI and large prosthesis size on DV/kg might be explained by the effect of the patients' weight on the outcome measurement and bleeding surface from bony cut in TKR. The patients with larger surface of bony cut from TKR (larger prosthesis size) resulted in greater bleeding surface from bony cut (greater DV/kg). Therefore, the patients with same size of bleeding surface and lower BMI would result in higher DV/kg than those who had higher BMI.

The strength of this study was that our protocol included only the same IA-TXA method with only one surgeon using the same surgical technique, prosthesis type, and postoperative care. Moreover, to our best knowledge, this was the first study that has demonstrated the correlation between blood loss and transfusion and the perioperative factors after using IA-TXA method. Based on our results, we recommended using the specific blood preservative strategy in those patients with significant risk for postoperative transfusion (low preoperative Hb and low BMI), as the preoperative workup and treatment for anemia and malnutrition and/or the preoperative erythropoietin therapy [16, 29].

However, this study also had several limitations. First, this study was retrospective which might be inherently limited by the nature of the study and the completeness of patients' chart review. Secondly, our sample size was also relatively not large and might not be able to detect other significant predictive factors. Therefore, the multicentered prospective study with larger sample size should be performed to find out the other related factors. Lastly, although the IA-TXA application should be theoretically effective for reduction of blood loss and transfusion in TKR, this effect might not be the same, among the different regimens. Moreover, the application of IA-TXA method varied highly among the literature including the dosage, the application technique, and the use of drain clamp. Thus, the result of this study using a single low-dosage application together with 2-hour drain clamp might be different to the other methods.

## 5. Conclusion

This study showed that the patients undergoing primary TKR using IA-TXA application and having low preoperative Hb and BMI had a significant risk of postoperative transfusion. Moreover, multiple perioperative factors could result in higher PBL. Therefore, more specific strategy of blood loss preservation might be useful to address this group of patients.

## Figures and Tables

**Table 1:** Characteristics of the 299 TKRs underwent primary TKR with IA-TXA application during 2013-2014 and risk factors for receiving blood transfusion.

Patients characteristics	Total (*n* = 299)	BT		*p* value
		Received BT (*n* = 16)	No BT (*n* = 283)	
Age, year +	69 ± 8	71 ± 9	69 ± 8	0.57
Male gender ■	54 (18)	3 (19)	51 (18)	1.00
Right side ■	145 (48)	10 (63)	135 (48)	0.31
Weight, kg +	65 ± 12	59 ± 13	66 ± 12	0.04^**∗**^
Height, cm +	156 ± 8	157 ± 8	155 ± 8	0.48
BMI, kg/m2 +	27.0 ± 4.3	24.0 ± 4.4	27.2 ± 4.3	0.007^**∗**^
ASA grading ■				
1-2	127 (42)	6 (37)	121 (43)	0.80
3-4	172 (58)	10 (63)	162 (57)	
CCI +	3.7 ± 1.0	3.8 ± 1.3	3.7 ± 1.0	0.63
Comorbidities ■				
HT	230 (77)	12 (75)	218 (77)	0.79
RA	9 (3)	0 (0)	9 (3)	1.00
Antiplatelet agents usage ■	35 (12)	2 (13)	33 (12)	1.00
OA KL grade 4 ■	197 (67)	12 (75)	185 (65)	0.59
Femorotibial angle, degree +	−5.8 ± 6.4	−6.6 ± 5.9	−5.8 ± 6.4	0.65
Preoperative ROM, degree +	109 ± 19	118 ± 19	109 ± 19	0.08
Preoperative laboratory values +				
Hb, g/dL	12.6 ± 1.2	11.1 ± 1.3	12.6 ± 1.1	**<0.000**1^**∗**^
Platelet count, ×103/mm3	273 ± 80	256 ± 68	274 ± 81	0.40
aPTT, second	27.0 ± 2.4	26.8 ± 2.6	27.1 ± 2.4	0.74
INR	1.01 ± 0.06	1.05 ± 0.10	1.01 ± 0.05	0.02^**∗**^
Albumin, g/dL	38.7 ± 3.6	36.8 ± 5.3	38.8 ± 3.5	0.05^**∗**^
CrCl, ml/min	70.6 ± 23.4	58.1 ± 19.2	71.3 ± 23.4	0.04^**∗**^
General anesthesia ■	16 (5)	1 (6)	15 (5)	0.59
Operative time, minute +	80 ± 17	72 ± 9	81 ± 17	0.06
Large prosthesis size ■	85 (28)	4 (25)	81 (29)	1.00
IBL, mL +	47 ± 35	47 ± 36	44 ± 15	0.74
IBL/kg, mL/kg +	0.7 ± 0.5	0.7 ± 0.5	0.8 ± 0.3	0.67
DV, mL +	404 ± 225	550 ± 320	396 ± 216	0.01^**∗**^
DV/kg, mL/kg +	6.3 ± 3.5	9.5 ± 3.2	6.1 ± 3.3	0.0005^**∗**^
THL, g/dL +	1.6 ± 0.5	2.3 ± 1.5	1.6 ± 0.9	0.008^**∗**^
ETBL, mL +	196 ± 122	495 ± 136	179 ± 96	**<0.000**1^**∗**^

BT: blood transfusion; +: value presented as mean ± standard deviation; ■: value presented as number of cases (percentage); BMI: body mass index; ASA: American Society of Anesthesiologists; CCI: Charlson Comorbidity Index; HT: hypertension; RA: rheumatoid arthritis; OA: osteoarthritis; KL: Kellgren-Lawrence; ROM: range-of-motion; Hb: hemoglobin; aPTT: activated partial thromboplastin time; INR: international normalized ratio; CrCl: creatinine clearance; IBL: intraoperative blood loss; IBL/kg: intraoperative blood loss per kg; DV: drainage volume; DV/kg: drainage volume per kg; *∗*: significant value with *p* < 0.05.

**Table 2:** Univariate regression analysis to determine predictors (*p* < 0.15) of allogeneic blood transfusion and each type of excessive postoperative blood loss in the study patients.

Variables	BT			THL > 2.7 mg/dL			ETBL > 348.6 mL			DV/kg > 10.6 mL/kg		
	Odds ratio	95% CI	*p* value	Odds ratio	95% CI	*p* value	Odds ratio	95% CI	*p* value	Odds ratio	95% CI	*p* value
Age	1.021	0.955 to 1.091	0.54	0.996	0.949 to 1.046	0.879	0.959	0.916 to 1.005	0.08^*♯*^	1.019	0.969 to 1.070	0.47
Male gender	1.050	0.289 to 3.819	0.94	1.207	0.466 to 3.123	0.699	5.009	2.269 to 11.060	0.0001^*♯*^	2.250	0.962 to 5.261	0.06^*♯*^
Right side	1.827	0.647 to 5.163	0.26	0.727	0.335 to 1.581	0.421	0.794	0.371 to 1.698	0.55	0.849	0.393 to 1.834	0.68
Weight	0.948	0.903 to 0.996	0.03	0.986	0.953 to 1.019	0.394	1.060	1.027 to 1.093	0.0003^*♯*^	0.946	0.911 to 0.983	0.004^*♯*^
Height	1.025	0.962 to 1.099	0.45	0.997	0.947 to 1.049	0.893	1.119	1.065 to 1.175	**<0.000**1^*♯*^	1.010	0.961 to 1.061	0.70
BMI	0.809	0.701 to 0.934	0.004^*♯*^	0.964	0.879 to 1.057	0.437	1.052	0.969 to 1.143	0.22	0.811	0.725 to 0.907	0.0002^*♯*^
Antiplatelet agent use	1.082	0.235 to 4.975	0.92	0.532	0.121 to 2.341	0.404	0.238	0.031 to 1.807	0.17	0.858	0.246 to 2.998	0.81
ASA physical status grade 3-4	1.245	0.440 to 3.519	0.68	0.771	0.358 to 1.661	0.507	0.615	0.288 to 1.311	0.21	0.771	0.358 to 1.661	0.51
CCI	1.141	0.694 to 1.875	0.60	0.931	0.634 to 1.367	0.714	0.811	0.552 to 1.191	0.29	0.895	0.609 to 1.317	0.57
Comorbidities												
HT	0.895	0.279 to 2.868	0.85	1.168	0.455 to 2.992	0.748	0.670	0.292 to 1.539	0.35	0.937	0.382 to 2.296	0.89
RA	n/a			n/a			n/a			n/a		0.99
Femorotibial angle	0.980	0.901 to 1.066	0.64	1.01	0.953 to 1.071	0.734	0.983	0.924 to 1.046	0.58	0.986	0.927 to 1.050	0.67
OA KL grade 4	1.589	0.499 to 5.058	0.43	0.982	0.439 to 2.199	0.960	0.883	0.403 to 1.935	0.76	1.402	0.598 to 3.287	0.44
Preoperative ROM	1.028	0.998 to 1.060	0.07^*♯*^	1.007	0.987 to 1.028	0.490	1.024	1.002 to 1.046	0.03^*♯*^	1.021	0.999 to 1.044	0.06^*♯*^
Preoperative laboratory values												
Hb	0.257	0.144 to 0.459	**<0.000**1^*♯*^	2.226	1.556 to 3.184	**<0.000**1^*♯*^	1.894	1.355 to 2.647	0.0002^*♯*^	1.200	0.872 to 1.652	0.26
Platelet count	0.997	0.990 to 1.004	0.37	1.003	0.999 to 1.008	0.118^*♯*^	0.999	0.994 to 1.004	0.69	0.995	0.990 to 1.001	0.09^*♯*^
aPTT	0.963	0.779 to 1.190	0.73	0.967	0.823 to 1.135	0.678	1.044	0.895 to 1.217	0.59	0.974	0.830 to 1.144	0.75
INR	26313.398	14.063 to 49234185.914	0.008^*♯*^	0.182	0.000 to 156.350	0.621	0.476	0.001 to 335.973	0.82	0.231	0.000 to 193.500	0.67
CrCl	0.969	0.942 to 0.996	0.03^*♯*^	1.003	0.987 to 1.019	0.765	1.026	1.011 to 1.040	0.0006^*♯*^	0.981	0.963 to 1.001	0.06^*♯*^
Albumin	0.867	0.759 to 0.989	0.03^*♯*^	0.992	0.892 to 1.102	0.877	0.969	0.874 to 1.075	0.55	1.116	0.998 to 1.247	0.05^*♯*^
General anesthesia	1.191	0.147 to 9.631	0.87	1.355	0.292 to 6.279	0.698	1.301	0.281 to 6.021	0.74	1.355	0.292 to 6.279	0.70
Operative time	0.965	0.931 to 1.001	0.05^*♯*^	0.998	0.975 to 1.021	0.838	0.990	0.968 to 1.014	0.41	0.998	0.975 to 1.021	0.84
Large prosthesis size	0.831	0.260 to 2.653	0.76	1.368	0.608 to 3.078	0.448	4.522	2.071 to 9.875	0.0002^*♯*^	2.616	1.203 to 5.690	0.02^*♯*^

BT: blood transfusion; THL: total hemoglobin loss; ETBL: estimated total blood loss; RD/kg: drainage volume per kg; CI: confidence interval.

BMI: body mass index; ASA: American Society of Anesthesiologist; CCI: Charlson Comorbidity Index,

HT: hypertension; RA: Rheumatoid arthritis; OA: osteoarthritis; KL: Kellgren-Lawrence; ROM: range-of-motion,

Hb: hemoglobin; aPTT: activated partial thromboplastin time; INR: international normalized ratio; CrCl: creatinine clearance.

*♯*: significant value with *p* < 0.15.

**Table 3:** Significant factors for allogeneic blood transfusion and excessive postoperative blood loss from multivariate logistic regression analysis.

Significant factors	OR	95% CI	*p* value
*BT*			
Hb	0.23	0.12 to 0.43	<0.0001
BMI	0.77	0.65 to 0.91	0.003
*THL > 2.7 g/dL*			
Hb	2.23	1.56 to 3.18	<0.0001
*ETBL > 348.6 mL*			
Height	1.11	1.05 to 1.17	0.0002
Hb	1.65	1.15 to 2.39	0.007
Preoperative ROM	1.03	1.00 to 1.06	0.02
CrCl	1.02	1.01 to 1.04	0.006
*DV/kg > 10.6 mL/kg*			
BMI	0.78	0.69 to 0.88	0.0001
Large prosthesis size	3.91	1.67 to 9.14	0.002

BT: blood transfusion; THL: total hemoglobin loss; ETBL: estimated total blood loss; DV/kg: drainage volume per kg.

Hb: hemoglobin; BMI: body mass index; ROM: range-of-motion; CrCl: creatinine clearance.

## List of Abbreviations

IA-TXA: Intra-articular tranexamic acid
PBL: Perioperative blood loss
TKR: Total knee replacement
BT: Blood transfusion
THL: Total hemoglobin loss
ETBL: Estimated total blood loss
DV/kg: Drainage volume per kg
Hb: Hemoglobin
BMI: Body mass index
TXA: Tranexamic acid
OA: Osteoarthritis
KL: Kellgren and Lawrence
ROM: Range-of-motion
ASA: American Society of Anesthesiologists
aPTT: Activated partial prothrombin time
INR: International normalized ratio
CrCl: Creatinine clearance
CCI: Charlson Comorbidity Index.
